# METTL1 promotes colorectal cancer cell proliferation by attenuating CHEK2-induced G1/S phase arrest

**DOI:** 10.1016/j.gendis.2023.04.011

**Published:** 2023-05-18

**Authors:** Houxiang Jiang, Ying Liu, Huibin Song, Jinquan Xia, Ying Tian, Luolin Wang, Mingwei Li, Zhenyu Xu, Zhenglei Xu, Chang Zou

**Affiliations:** a Department of Gastrointestinal Surgery, The First Affiliated Hospital of Wannan Medical College (Yijishan Hospital of Wannan Medical College), Wuhu, Anhui 241001, China; b Anhui Province Clinical Research Center for Critical Respiratory Medicine, Wuhu, Anhui 241001, China; c Department of Clinical Medical Research Center, The Second Clinical Medical College (Shenzhen People's Hospital) of Jinan University, The First Affiliated Hospital of Southern University of Science and Technology, Shenzhen, Guangdong 518020, China; d Integrated Chinese and Western Medicine Postdoctoral Research Station, Jinan University, Guangzhou, Guangdong 510632, China; e School of Pharmacy, Southwest Medical University, Luzhou, Sichuan 646000, China; f Department of Gastroenterology, Shenzhen People's Hospital (The Second Clinical Medical College, Jinan University; The First Affiliated Hospital, Southern University of Science and Technology), Shenzhen, Guangdong 518020, China; g Precision Medicine Center, The First Affiliated Hospital of Wannan Medical College (Yijishan Hospital of Wannan Medical College), Wuhu, Anhui 241001, China; h School of Medicine, Life and Health Sciences, The Chinese University of Hong Kong, Shenzhen, Guangdong 518172, China

Colorectal carcinoma (CRC), the third most commonly diagnosed cancer, accounts for 9.7% of all newly diagnosed cancer cases and 9.4% of cancer-related deaths globally.^1^ Recent studies have demonstrated that post-transcriptional RNA modifications, such as N^6^-methyladenosine, N^5^-methylcytosine, and N^7^-methylguanosine, play critical roles in the regulation of mRNA stability and translation, primary microRNA processing, and lncRNA-protein complex that contributes to the progression of human cancer.^2^^,^^3^ Here, we found that the expression of the member of methyltransferase-like (METTL) family-METTL1, the m^7^G “writers”, was remarkably up-regulated in colorectal cancer tissue and positively correlated with poor prognosis. METTL1 knockdown suppressed colorectal cancer cell growth and G1/S phase transition. Further functional experiments indicate that METTL1 could directly interact with checkpoint kinase 2 (CHEK2) and suppress its protein expression, which was abrogated by BML-277, the CHEK2 inhibitor. Our data uncover that METTL1 plays an important supportive role in colorectal cancer proliferation and progression, providing a potential therapeutic target for colorectal cancer.

We first analyzed the expression profiles of METTL family genes using the data from The Cancer Genome Atlas database and single-cell sequencing (GSE196006) (Table S1). The results indicated that METTL1 was remarkably up-regulated in colorectal cancer tissues compared with normal tissues (Fig. 1A, B). Immunohistochemistry staining was further used to validate the expression of METTL1 by tissue micro-array analysis for a large cohort with 93 tumor tissues and 87 non-tumor tissues. The result showed that METTL1 expression was up-regulated in colorectal cancer tissues compared to matched adjacent normal intestinal epithelial tissues (Fig. S1 and Table S2). Kaplan–Meier analysis showed that the elevated METTL1 expression was significantly positively correlated with shortened overall survival of colorectal cancer patients (*P* = 0.017) (Fig. 1C).

**Figure 1:**
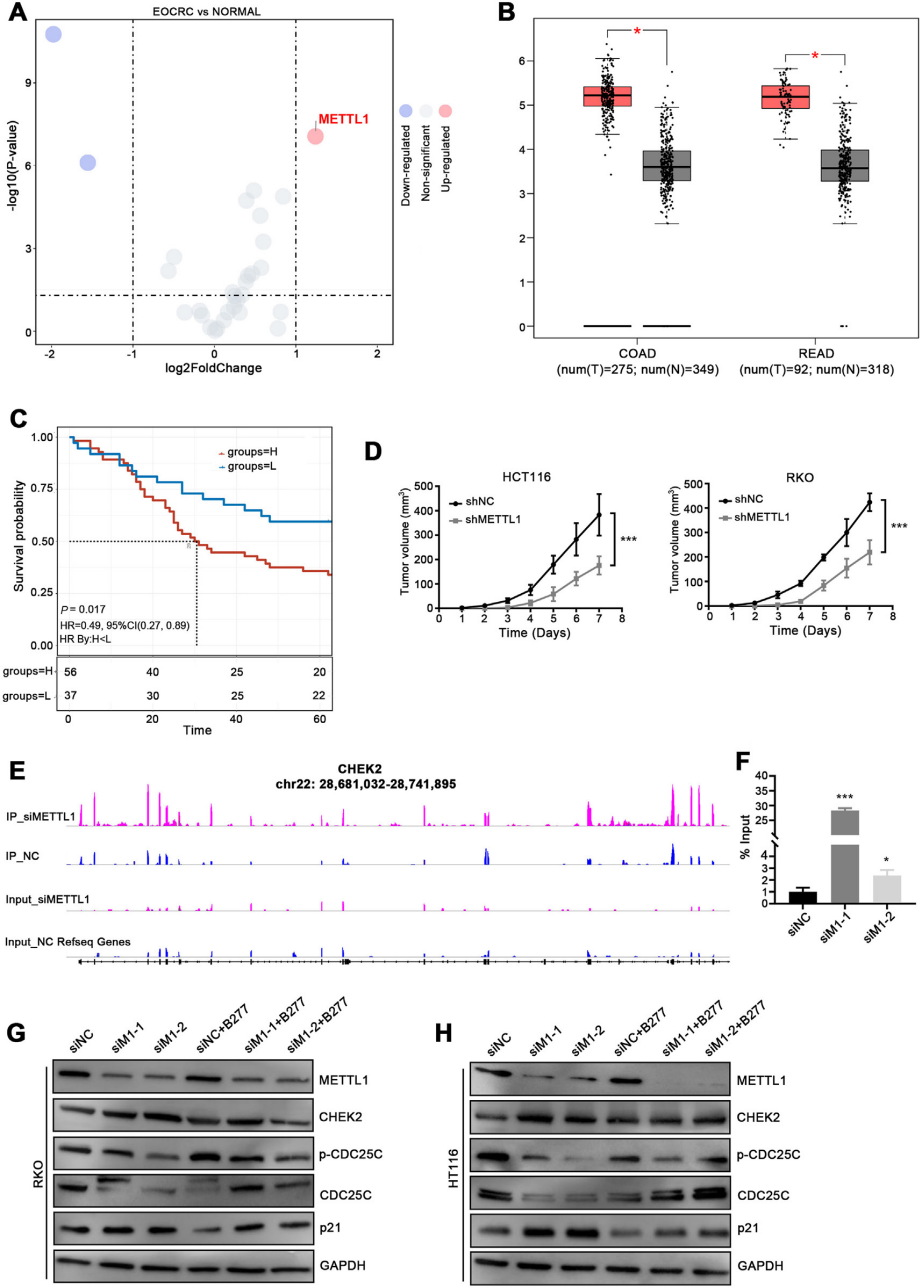
METTL1 promoted colorectal cancer cell progression by down-regulating CHEK2. **(A)** The mRNA expression profile of METTL family genes from single-cell sequencing (GSE196006). **(B)** The relative expression of METTL1 from Gene Expression Profiling Interactive Analysis database. **(C)** Kaplan–Meier survival curve analysis of CRC patients with high or low METTL1 expression. **(D)** ShMETTL1 and shNC HCT116 cells were subcutaneously injected into BALB/c-nude mice. Seven days after cell inoculation, the tumor was measured twice a week. Tumor volumes were calculated. **(E)** RIP-seq in METTL1-suppressed HCT116 cells showed the interaction between METTL1 and CHEK2. **(F)** RIP-qPCR analysis of CHEK2 in METTL1-suppressed HCT116 cells. **(G)** After treatment with BML-277 (40 nM) for 24 h, followed by transfection for 4 h, cells were collected and detected by Western blot. All values are the average of triplicate experiments with standard deviation indicated by the error bars. ^∗^*P* < 0.05, ^∗∗∗^*P* < 0.001.

To explore the biological functions of METTL1 in the progression of colon cancer, we overexpressed and inhibited the expression of METTL1 in HCT116 and RKO cell lines. The results showed that METTL1 promoted CRC cell growth and cell cycle progression (Fig. S2, 3). The impact of METTL1 was further investigated in BALB/c nude mice who were subcutaneously inoculated with HCT116 and RKO cells stably expressing shMETTL1 or shNC. The tumor growth was remarkably inhibited in mice bearing shMETTL1, with a striking decrease in tumor size (Fig. 1D). The lowered expression of Ki67 from immunohistochemistry staining was also observed in shMETTL1 xenograft tumors (Fig. S4), suggesting decreased cell proliferation of METTL1 knockdown xenograft tumors compared with control tumors. Taken together, these findings indicated that METTL1 promoted colon cancer cell proliferation both *in vitro* and *in vivo*.

Global gene expressions of siMETTL1-1, siMETTL1-2, and siNC cells were sequenced to explore the mechanism of METTL1 on colon cancer development. A total of 3701 differentially expressed genes were identified with 1572 up-regulated genes and 2129 down-regulated genes (Fig. S5A). Kyoto Encyclopedia of Genes and Genomes pathway enrichment analysis showed that METTL1 participated in cell cycle regulation (Fig. S5B), with the significantly up-regulated cell cycle-related gene CHEK2 in siMETTL1 cells, which mutations were detected in several familial cancers^4^ (Fig. S5C, D). Moreover, the protein levels of CHEK2 and its downstream genes were detected by Western blot assays.^5^ The results showed that the protein expressions of CHEK2 and p21 were significantly induced in METTL1-suppressed HCT116 and RKO cells, accompanied by decreased expression levels of CDC25C and p-CDC25C (Fig. S5E). The Western blot results in METTL1-overexpressed HCT116 and RKO cells were in line with those in corresponding METTL1 knockdown cells (Fig. S5F). Therefore, the results indicated that METTL1 could promote CRC cell cycle progression, with the down-regulated level of CHEK2.

Given that METTL1 could promote CRC cell proliferation and G1/S transition and inhibit the expression level of CHEK2, we further evaluated whether METTL1-induced cell proliferation and G1/S translation were CHEK2-dependent. The results of RIP-seq in METTL1-suppressed RKO cells showed a direct interaction between METTL1 and CHEK2, which was validated by RIP-qPCR (Fig. 1E, F). BML-277, an ATP-competitive inhibitor of CHEK2, was used in METTL1-suppressed HCT-116 cells. The results showed BML-277 treatment (40 nM) promoted cell proliferation and G1/S transition which was reduced by METTL1 inhibition in HCT116 cells (Fig. S6A–C). Furthermore, increased p21 protein level and decreased CDC25C and p-CDC25C protein levels were all abrogated by treatment of BML-277 in HCT116 cells (Fig. 1G). Taken together, our results supported that METTL1 promoted CRC cell proliferation and G1/S translation in a CHEK2-dependent manner.

## Author contributions

ZX and CZ designed the research. HJ, YL, HS, ZX, and CZ wrote and revised the manuscript. HJ, YL, HS, JX, YT, LW, ML, and ZX performed the experiments. All authors contributed to the manuscript and approved the submitted version.

## Conflict of interests

The authors declare no conflict of interests.

## Funding

This work was supported by the Science and Technology Foundation of Shenzhen, Guangdong, China (No. JCYJ20210324115800001), the Natural Science Foundation of Guangdong Province, China (No. 2018A0303130278), the Basic and Applied Basic Research of Guangdong Province, China (No. 2021A1515110253), and the Shenzhen Key Medical Discipline Construction Fund (China) (No. SZXK053).
