# Supplementary material for: METTL1 promotes colorectal cancer cell proliferation by attenuating CHEK2-induced G1/S phase arrest
Source: Genes Dis. 2023 May 18;11(2):579–81. doi: 10.1016/j.gendis.2023.04.011 (PMC10491903; doi:10.1016/j.gendis.2023.04.011)
Supplement: Multimedia component 1 [file mmc1.doc]

**Supplementary Data**

**Supplementary materials and methods**

**Methyltransferase-like gene family expression analysis and tissue samples**

The methyltransferase-like gene family expression profiles in cancer tissues and normal tissues were obtained from The Cancer Genome Atlas dataset, including all existing cancer cases. The results were visualized with R package. A tissue array containing a total of 93 colon cancer samples and matched adjacent normal tissues with follow-up data was obtained from Shanghai Outdo Biotech Co. Ltd. (Shanghai, China). Immunohistochemistry assays were performed on the tissue microarray chip according to standard protocols provided by Thermo Scientific. Briefly, sections were incubated with anti-METTL1 antibody (Thermo, Cat#PA5-80810, 1:200 dilution) 2 h at 37 ℃, and subsequently incubated with streptavidin-conjugated horseradish peroxidase. Sections were visualized with a 3, 3-diaminobenzidaine kit. All imunohistochemistry samples were assessed by two independent pathologists blinded to both the sample origins and the subject outcomes. The tissue microarray was scanned using Sanscope XT (Aperio, Shanghai, China). The clinical features of the patients are listed in Table S2. For survival analyses, patient overall survivals stratified by expression of METTL1 were presented as the Kaplan-Meier plots and tested for significance using log-rank tests.

**Immunohistochemistry**

The tissue microarray and tissue sections were cultured overnight with Anti-METTL1 (Proteintech, Cat#14994-1-AP), Anti-Ki67 (CST, Cat#9449), Anti-CHEK2 (CST, Cat#3440) at 4 ℃ and then cultured with secondary antibody and horseradish peroxidase. The imunohistochemistry staining and evaluation were performed according to the previous report^1^.

**Cell lines and cultures**

Colon cancer (CC) cell lines (HCT116, RKO) were purchased from American Type Culture Collection. HCT116 were cultured in RPMI-1640 medium (Gibco Company, USA) containing 10% fetal bovine serum (FBS, Gibco Company, USA) and RKO were cultured in the Dulbecco`s modified Eagle medium (DMEM, Gibco Company, USA) containing 10% FBS (Gibco Company, USA). These cells were cultured under the standard conditions with a humidified atmosphere containing 5% CO_2_ at 37 ℃. The identity of HCT116 and RKO cell lines was confirmed by genetic profiling using short tandem repeat loci by Cellcook Biotech Corporation (Guangzhou, China) and IGE Biotechnology LTD (Guangzhou, China). All of the cell lines were confirmed with negative mycoplasma contamination.

**RNA interference and overexpression**

METTL1 small inhibitory RNA (siMETTL1-1: 5’-CCAGCCAUCUUCCGAAGAATT-3’, siMETTL1-2: 5’-GCAGUGACCUCCCAAACCATT-3’) and a negative control siRNA (NC: 5’-UUCUCCGAACGUGUCACGUTT-3’) were chemically synthesized by GenePharma (Shanghai, China). METTL1 overexpression plasmid (pENTER-METTL1, NM_005371.6) was purchased from WZ Biosciences Corporation (Shandong, China). HCT116 and RKO cells were grown in 6-well plates at 2×10^5^ cells/well 36 h earlier before transfection. Upon 60% confluence, the cells were added with the serum-free medium for 1 h. The siRNA (100 nmol/L) or plasmid (2 μg) was transfected into cells with lipofectamine 3000 reagents (Thermo, USA).

**RNA extraction and real-time quantitative polymerase chain reaction (qPCR)**

Total RNA was extracted from cells using TRIZOL Reagent (Invitrogen, USA), and cDNA was synthesized from 1 μg RNA with an RT Reagent kit (Takara Bio, Japan) as recommended by the manufacture. Real-time quantitative PCR reactions for the quantification of gene expression were performed with Bio-Rad iQ5 Real-Time PCR System. The primers sequences used in this study were listed in Table S3.

**Western blotting and antibodies**

Cells were lysed with ice-cold RIPA lysis and extraction buffer (Thermo, Cat#89901) with a protease and inhibitor tablet (Roche, Cat#38681900), and protein concentration was determined with the BCA Protein Assay Kit (Pierce, Rockford, IL, USA). Proteins (20 μg) were resolved on 12.5% polyacrylamide gel, transferred onto Immuno-Blot PVDF membranes (Millipore Corp, Atlanta, GA, US). The membranes were blocked in 5% non-fat milk powder at room temperature for 1 h, and then incubated at 4 ℃ with primary antibodies overnight: anti-METTL1 (ABclonal, Cat#A16651), anti-GAPDH (CST, Cat#5174S), anti-CHEK2 (CST, Cat#3440), anti-p21 (CST, Cat#2947S), anti-p-CDC25C (ser216) (CST, Cat#4901T), anti-CDC25C (ABclonal, Cat#A1672). All antibodies were diluted in 1:1000 with 5% BSA. Membranes were washed three times with TBST and further incubated with HRP-conjugated secondary antibodies (CST, Cat#7074V) in 5% milk (TBST) at room temperature for 1 h. All blots were developed using enhanced chemiluminescence (Thermo, Cat#34580).

**Cell proliferation assay**

Cell Counting Kit-8 (MedChem Express, Monmouth Junction, NJ, USA) was utilized to evaluate cell proliferation according to the manufacture’s recommendations. Briefly, cells transfected with METTL1 siRNA or overexpression plasmid were seeded in a 96-well plate. Cell proliferation was documented every 24 h for 3 days. The number of viable cells was assessed by measurement of the absorbance at 450 nm using Cytation 5 (BIO-TEK Instruments, Winooski, VT, USA).

**Colony formation assay**

Cells were seeded at a density of 1.5×10^3^ cells/well in six-well plates. After incubation at 37 ℃ for 14 days, colonies were fixed with 4% paraformaldehyde for 15 min and stained by crystal violet for 30 min at room temperature. Then plates were washed with water and photographed with Cytation5 (BIO-TEK Instruments, Winooski, VT, USA). Macroscopic colonies of each well were counted.

**Flow cytometry assays**

The cell cycle was assessed by FCM. The harvested cells were washed twice with FBS and then fixed in a 70% ethanol solution overnight at 4 ℃. The fixed cells were then washed once with PBS and re-suspended as a single-cell suspension. The suspension was incubated with 0.5 mL of PI (50 μg/mL) for 30 min at 37 ℃ and assessed using BD FACSCaliburTM (Becton Dickinson, USA).

**Xenograft tumor model**

The animal study was approved by the Jinan University Institutional Animal Care and Use Committee. HCT116 and RKO cell lines (1×10^7^ cells) pretreated with shNC or shMETTL1 vector were subcutaneously inoculated into Female BALB/c nu/nu mice (4-5 weeks old, n=4 in each group). Seven days after cell inoculation, tumor volume was measured twice a week and calculated with a formula (L×W^2^/2). The mice were sacrificed under general anesthesia with chloral hydrate (5%, 100 μL/10 g) on the 25 d and the tumors were removed, weighed, sectioned continuously. METTL1 shRNA was designed and synthesized by WZ Biosciences Corporation (Shandong, China). The sequence targeting human METTL1 was as follows: shMETTL1: GGTGTATACCATAACCGATGTTTCAAGAGAACATCGGTTATGGTATACACCTTTTTT.

**RNA-sequencing and data analysis**

The differential mRNA expression in siMETTL1-1, siMETTL1-2, and siNC was sequenced on Illumina HiSeq2500 by Frasergen Corporation (Wuhan, China) according to the manufacturer`s instructions. Approximately 83% reads were total mapped, while uniquely mapped were nearly 78%. The raw reads were aligned to the Home sapiens reference (GRCh38.p13) for mapping using HISAT2 (version 2.1.0). Differential expression genes were identified using DEseq2 between siNC group and siMETTL1 group under the criteria of Fold Change ≥ 1.5 or ≤ 0.5 and Benjamini & Hochberg-adjusted *p*-value ≤ 0.1. Genes with sequencing read count 1 in at least one comparison condition were used for the analysis. Kyoto Encyclopedia of Genes and Genomes analysis was performed using KOBAS. Plots were generated and the related statistics were conducted using R version 3.6.1.

**RNA-immunoprecipitation sequencing**

RIP was performed using the Magna RIP™ RNA-binding Protein Immunoprecipitation Kit (Millipore, Massachusetts, USA), according to the manufacturer’s protocol. Briefly, magnetic beads coated with 10 μg of Anti-METTL1 (Proteintech, Cat#14994-1-AP) were incubated with prepared indicated RKO cell lysates (SiNC and SiMETTL1) overnight at 4 °C. Washed RNA-protein complexes were treated with proteinase K digestion buffer. The co-precipitated RNAs were purified with phenol: chloroform: isoamyl alcohol and subsequently subjected to purity and concentration analysis using NanoDrop ND-1000. The cDNA was synthesized and sequenced on Illumina HiSeq2500 by Frasergen Corporation (Wuhan, China) according to the manufacturer`s instructions. Sequencing reads were aligned to the human genome GRCh38.p13 by HISAT2.

**Statistical analysis**

All data were expressed as mean ± standard deviation. SPAA 19.0 (SPSS, Chicago, IL) was employed to conduct all the statistical analyses. Statistical significance was determined by one-way ANOVA or by an unpaired two-tailed Student`s t-test. Chi-square test and Spearman`s rank test were used for correlation between METTL1 expression and clinicopathological characteristics. Survival analyses were plotted using Kaplan-Meier curves and compared using the log-rank test. Differences were considered significant when the *p*-value was less than 0.05.

**Supplementary references**

1. Zhang J, Song H, Chen C, et al. (2021). Methyltransferase-like protein 11A promotes migration of cervical cancer cells via up-regulating ELK3*. Pharmacol R*es. 2021;172:105814.

**Supplementary figures**

**Figure S1**


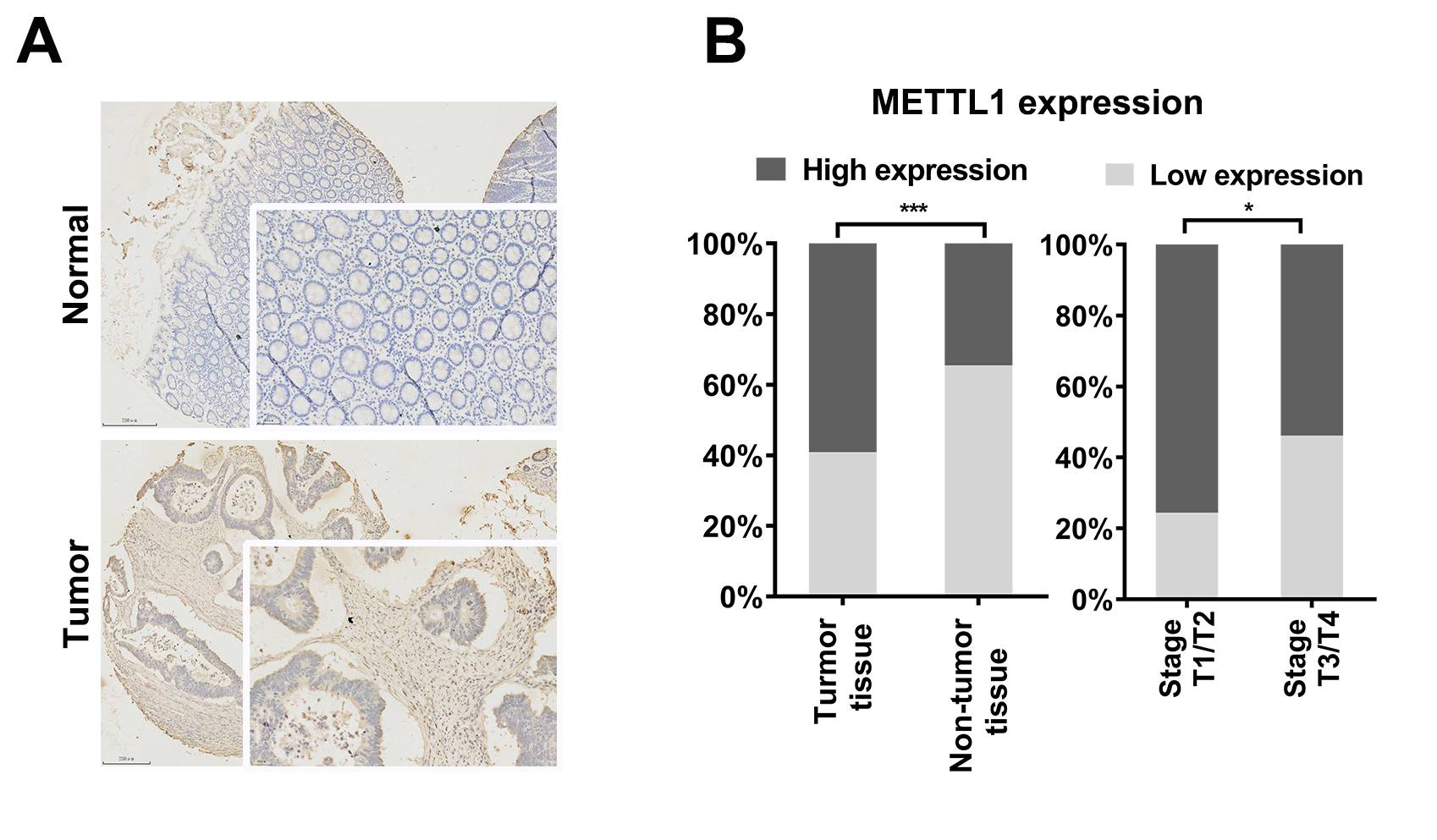


**Figure S1. METTL1 was up-regulated expressed in clinical colon cancer tissues.** (**A**) Immunohistochemical staining images of METTL1 in tissue micro-array analysis for a large cohort with 93 tumor tissues and 87 non-tumor tissues. (**B**) The METTL1 expression in colon cancer tissues and non-tumor tissues (left). The METTL1 expression in tumor tissue with different Tumor (T) stages (right).

**Figure S2**


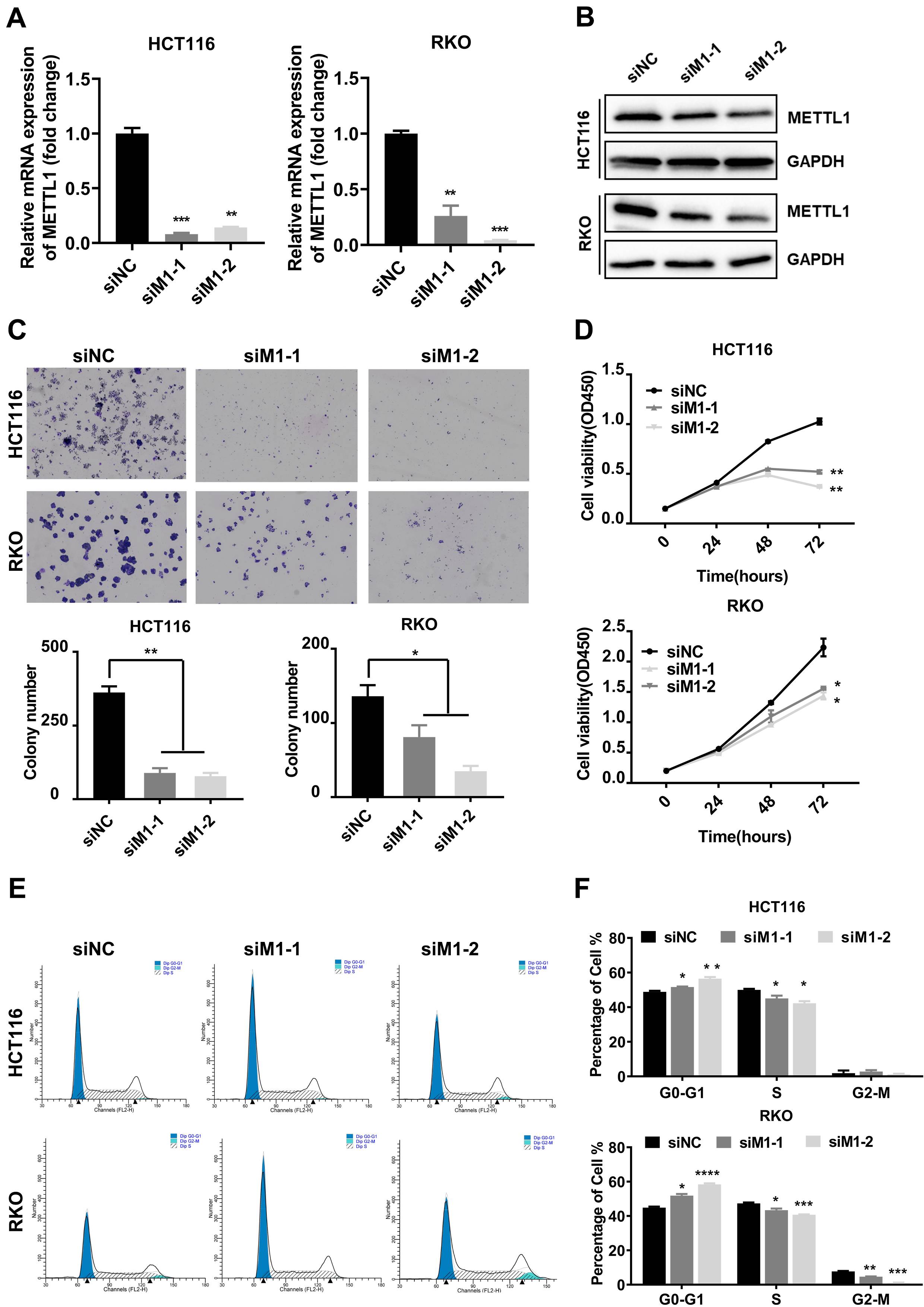


**Figure S2. Silencing of METTL1 inhibited the proliferation of colon cancer cells *in vitro*.** HCT116 and RKO cells were transfected with small interfering RNA (siMETTL1-1, siMETTL1-2) or negative control (siNC) mediated for 48 h. The knockdown efficiency was confirmed by qPCR (**A**) and Western blot (**B**). (**C**) Colony-formation assay performed with siMETTL1 or siNC cells. After siRNA treated for 24 h, cells were collected and seeded in six-well (1,000 cells/well) plate. After incubation for 14 days, colonies were stained by crystal violet and photographed. (**D**) Cell viability assay. After transfection for 24 h, cells were collected and seeded in 96-well plates (3,500 cells/well). CCK8 assay applied to measure the cell viability for 3 days. (**E**) Cell cycle analysis. All values are the average of triplicate experiments with SD indicated by the error bars. **p* < 0.05, ***p* < 0.01, ****p* < 0.001, *****p* < 0.0001.

**Figure S3**


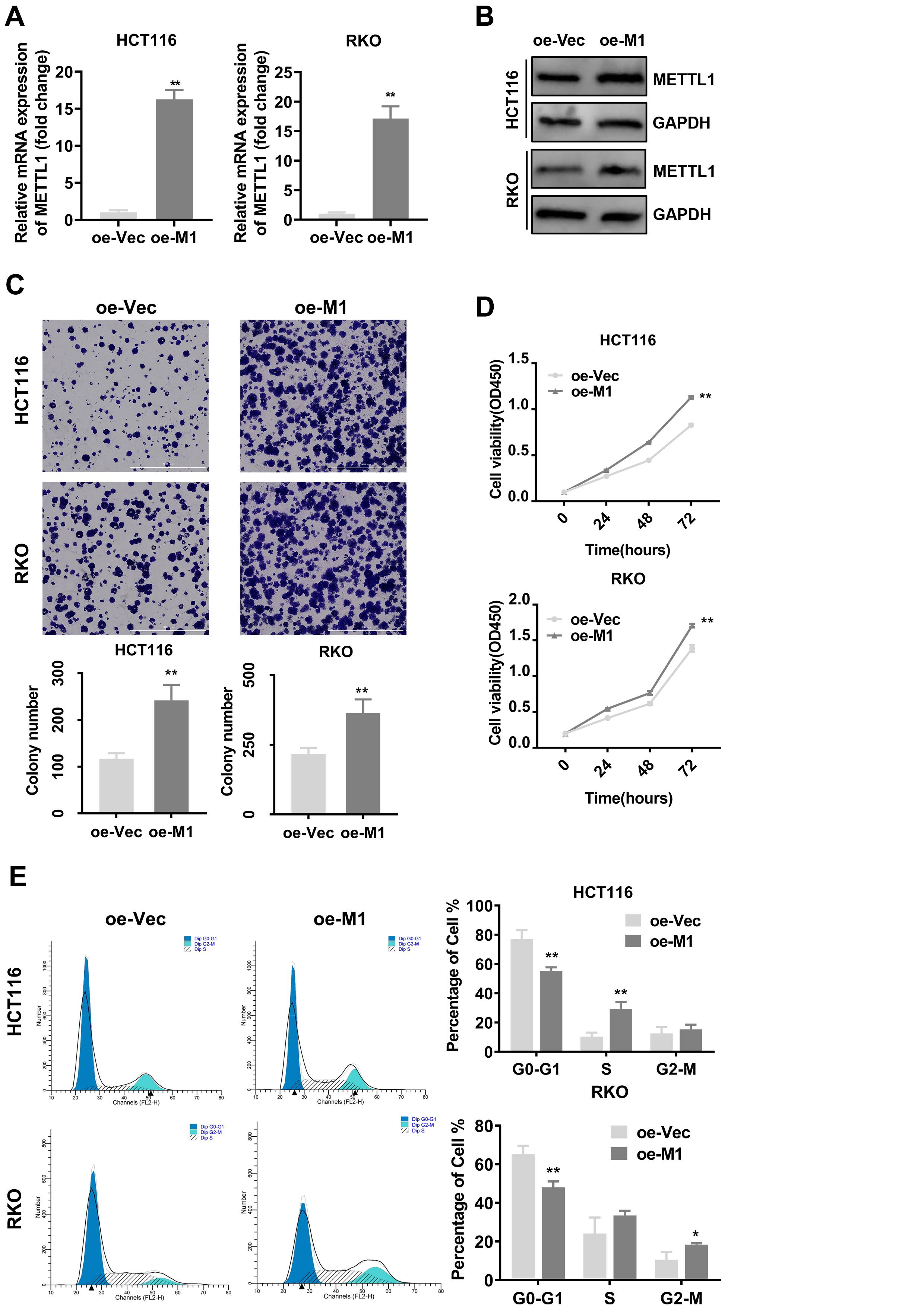


**Figure S3. Overexpression of METTL1 promoted the proliferation of colon cancer cells *in vitro*.** HCT116 and RKO cells were transfected with pENTER-METTL1 plasmid mediated for 48 h. The overexpression efficiency was confirmed by qPCR (**A**) and Western blot (**B**). (**C**) Colony-formation assay performed with vector or pENTER-METTL1. (**D**) Cell viability assay. (**E**) Cell cycle analysis. All values are the average of triplicate experiments with SD indicated by the error bars. **p* < 0.05, ***p* < 0.01.

**Figure S4**


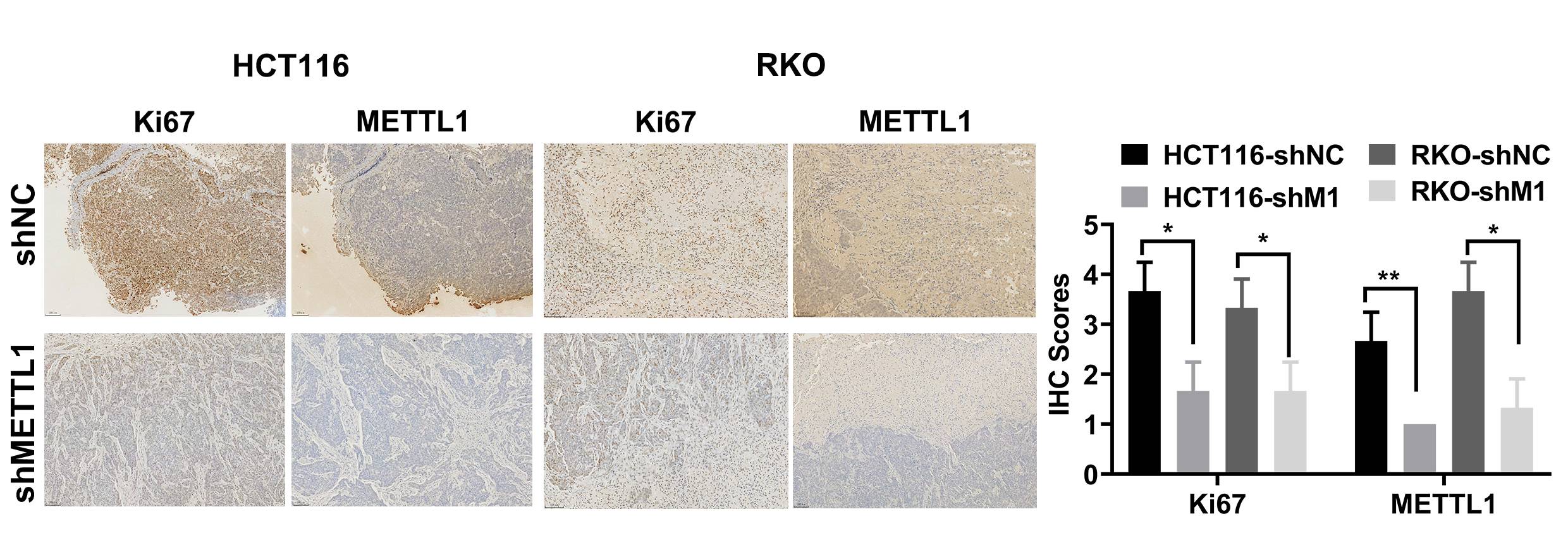


**Figure S4. METTL1 was essential for CRC cell growth *in vivo*.** ShMETTL1 and shNC HCT116 and RKO cells were subcutaneously injected into BALB/c-nude mice. Seven days after cell inoculation, tumor was measured twice a week. Tumor volumes were calculated. Immunohistochemical staining with indicated antibodies. Scale bar, 100 μm. Data represent mean ±SD, **p* < 0.05, ***p* < 0.01 and ****p* < 0.001 indicate significant difference between the groups.

**Figure S5**


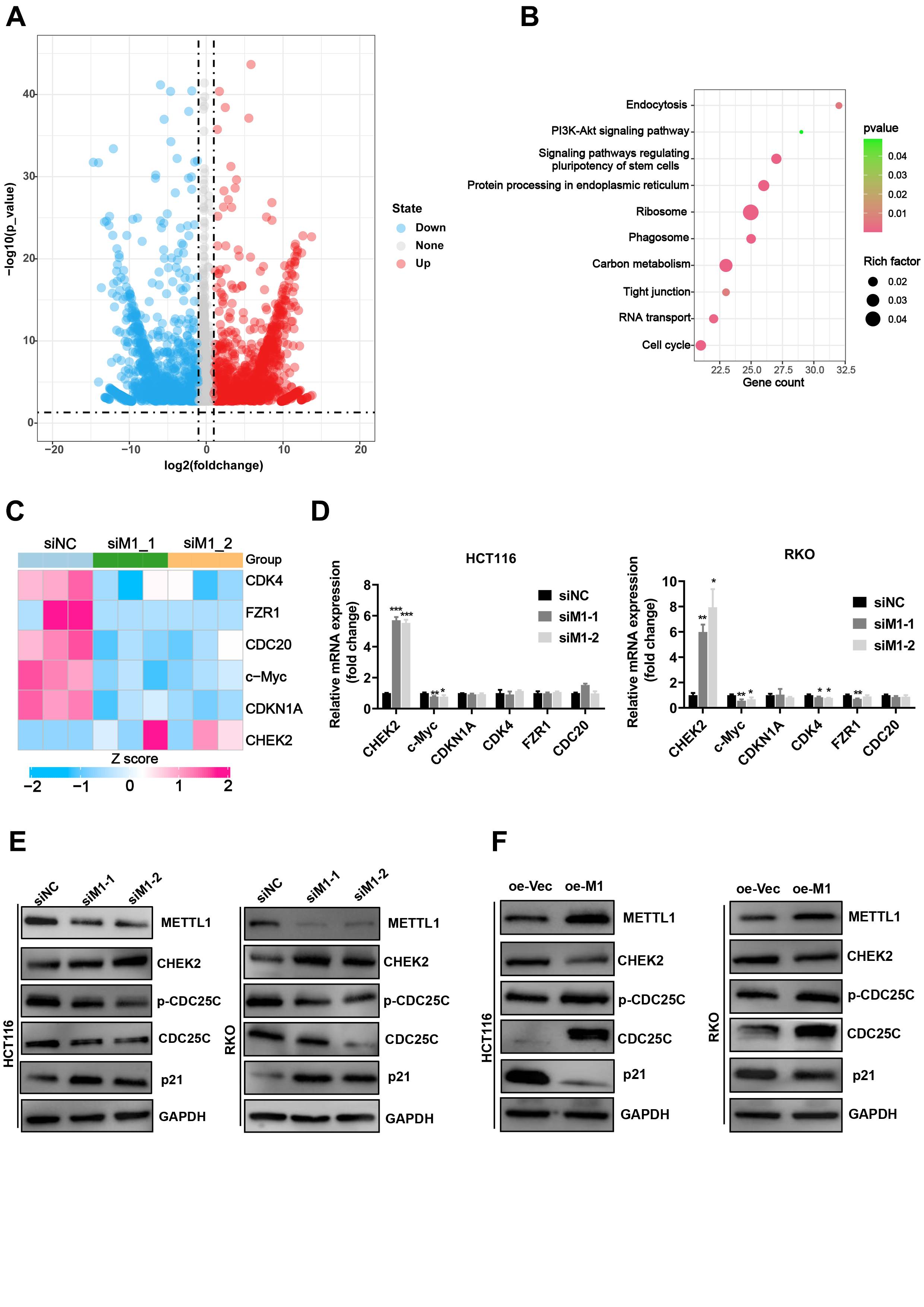


**Figure S5. METTL1 promoted colon cancer cell cycle progression through down-regulating CHEK2.** RNA-sequencing was used to analyze transcript profiles between siMETTL1 and siNC HCT116 cells. (**A**) Volcano Plot demonstrated the distribution the different expression genes between siMETTL1 group and siNC group. The X-axis represents the logarithm conversion of the fold difference to base 2 and the Y-axis represents the logarithm conversion of the corrected significant levels to base 10. Values with a FC > 2, and *p* < 0.05 are indicated in red and are considered significantly up-regulated; while values with a FC < -2 and *p* < 0.05 are indicated in green and are considered down-regulated. The gray dots represent genes with no significant differences. (**B**) Top ten descriptions of KEGG pathway analysis of different expression mRNAs shown in Dot Plot. Cell cycle related genes are significantly enriched with a *p*-value of 1.5e-4. (**C**) Heat map representation of transcriptome of the cell cycle-specific DEGs within siMETTL1 and siNC groups. (**D**) Cell cycle-specific DEGs were determined by real-time RT-PCR and normalized with GAPDH in HCT116 and RKO cells. (**E**) CHEK2 and its target genes expression were evaluated by western blot in siMETTL1 and siNC in HCT116 and RKO cells. (**F**) CHEK2 and its target genes expression were evaluated by western blot in OE-vector and OE-METTL1 in HCT116 and RKO cells. All values are the average of triplicate experiments with SD indicated by the error bars. **p* < 0.05, ***p* < 0.01, ****p* < 0.001.

**Figure S6**


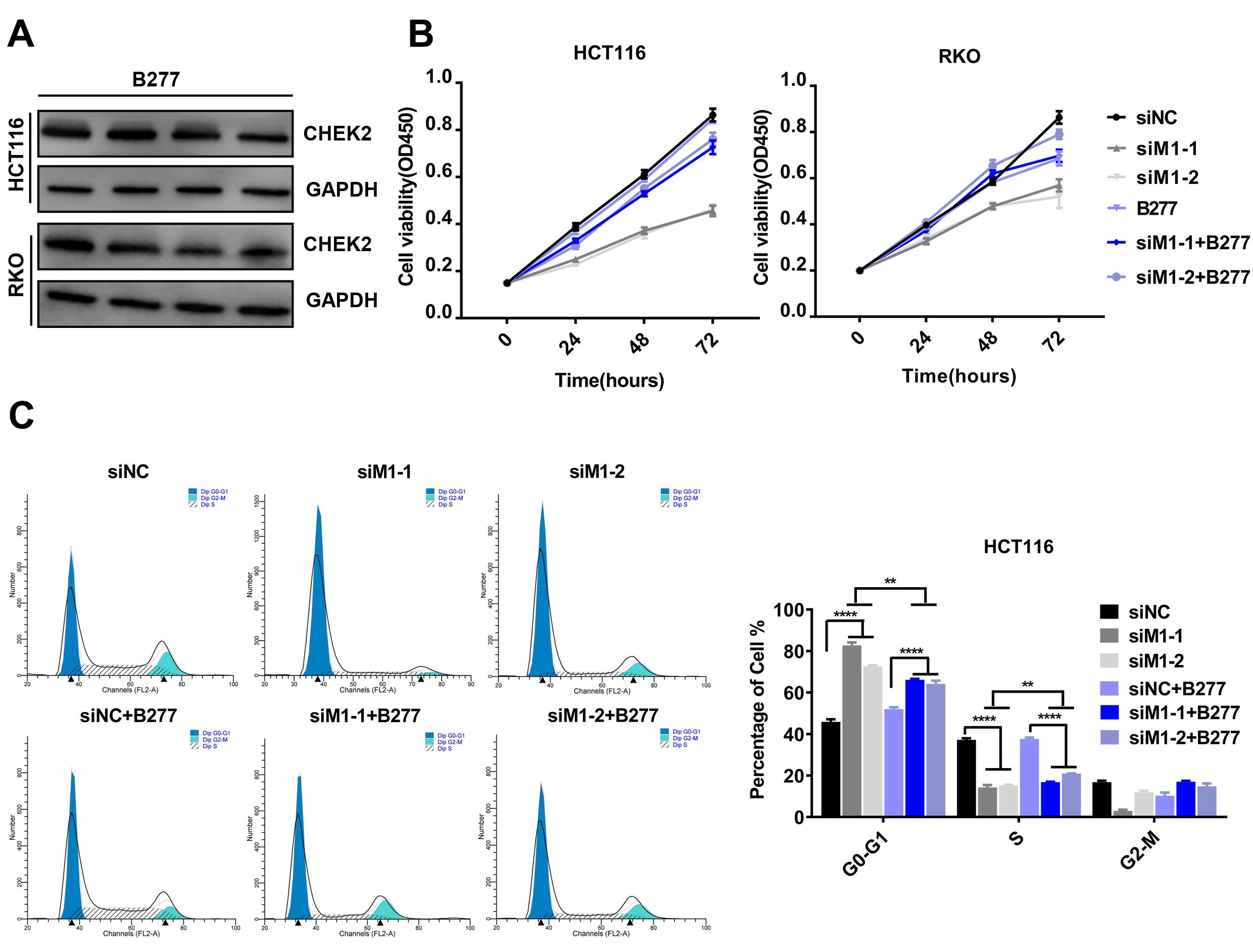


**Figure S6. Counteracting CHEK2 after METTL1 knock-down.** (**A**) Selection of working concentration of BML-277 in HCT116 and RKO cells with western blot. (**B**) Cell viability assay. After treatment with BML-277 (40 nM) for 24 h, following with transfection for 4 h, cells were collected and seeded in 96-well plates (3,500 cells/well). CCK8 assay applied to measure the cell viability for 72 h. (**C**) Cell cycle analysis. All values are the average of triplicate experiments with SD indicated by the error bars. ***p* < 0.01, *****p* < 0.0001.

**Table S1. The expression profiles of METTL family genes using the data from single-cell sequencing (GSE196006)**

| ID | EOCRC | NORMAL | baseMean | log_2_FoldChange | P value | P adj |
| --- | --- | --- | --- | --- | --- | --- |
| METTL7A | 924.717 | 2714.98 | 1819.848 | -1.977 | 1.75E-11 | 8.53E-10 |
| METTL1 | 334.812 | 131.784 | 233.298 | 1.241 | 8.67E-08 | 1.40E-06 |
| METTL24 | 18.741 | 64.846 | 41.793 | -1.555 | 7.81E-07 | 9.47E-06 |
| METTL13 | 801.449 | 553.442 | 677.446 | 0.484 | 8.00E-06 | 6.98E-05 |
| METTL5 | 1088.889 | 513.861 | 801.375 | 0.844 | 1.30E-05 | 0.0001065 |
| METTL2B | 406.869 | 299.186 | 353.028 | 0.392 | 1.84E-05 | 0.0001428 |
| METTL6 | 266.897 | 174.462 | 220.679 | 0.563 | 6.44E-05 | 0.0004184 |
| METTL21B | 114.919 | 78.606 | 96.762 | 0.6 | 0.0005771 | 0.002689 |
| METTL25 | 94.165 | 125.445 | 109.805 | -0.496 | 0.002025 | 0.00771 |
| METTL12 | 65.617 | 38.19 | 51.903 | 0.573 | 0.005037 | 0.01653 |
| METTL20 | 47.352 | 65.012 | 56.182 | -0.564 | 0.006534 | 0.02055 |
| METTL22 | 215.949 | 160.4 | 188.175 | 0.46 | 0.008143 | 0.02469 |
| METTL23 | 373.956 | 259.535 | 316.746 | 0.412 | 0.009872 | 0.02898 |
| METTL8 | 324.077 | 251.925 | 288.001 | 0.387 | 0.01586 | 0.04293 |
| METTL9 | 1878.876 | 1530.374 | 1704.625 | 0.224 | 0.03887 | 0.08937 |
| METTL3 | 648.05 | 510.704 | 579.377 | 0.34 | 0.04773 | 0.1053 |
| METTL2A | 473.465 | 376.889 | 425.177 | 0.249 | 0.05391 | 0.1158 |
| METTL21A | 271.301 | 207.263 | 239.282 | 0.283 | 0.07251 | 0.1464 |
| METTL18 | 167.444 | 133.368 | 150.406 | 0.24 | 0.1277 | 0.2285 |
| METTL14 | 452.867 | 494.095 | 473.481 | -0.181 | 0.1822 | 0.3001 |
| METTL21EP | 2.587 | 1.957 | 2.272 | 0.814 | 0.1991 | 0.3215 |
| METTL16 | 423.942 | 393.175 | 408.559 | 0.166 | 0.2034 | 0.3268 |
| METTL7B | 790.165 | 1209.14 | 999.652 | -0.365 | 0.208 | 0.3326 |
| METTL15P1 | 11.567 | 9.324 | 10.446 | 0.308 | 0.2101 | 0.3349 |
| METTL17 | 541.888 | 589.954 | 565.921 | -0.152 | 0.264 | 0.3971 |
| METTL4 | 208.737 | 206.276 | 207.506 | -0.05 | 0.7275 | 0.8176 |
| METTL15 | 257.327 | 242.419 | 249.873 | 0.033 | 0.8016 | 0.8712 |
| METTL10 | 81.79 | 79.439 | 80.615 | 0.008 | 0.9556 | 0.9722 |
| METTL11B | 1.839 | 0.15 | 0.994 | 0.779 | 0.7912 | 1 |
| METTL19 | 133.734 | 123.961 | 128.848 | 0.115 | 0.4056 | 0.5464 |

**Table S2 Correlation between METTL1 expression and clinicopathological characteristics**

|  | Variables | METTL1 expression | | Total | χ2 | p value |
| --- | --- | --- | --- | --- | --- | --- |
|  |  | Low | High |  |  |  |
| Age(year) | ≤60 | 12 | 22 | 34 | 0.6871 | 0.4071 |
|  | ＞60 | 26 | 33 | 59 |  |  |
| T stage | T1/T2 | 3 | 2 | 5 | 1.269 | 0.2599# |
|  | T3/T4a/b | 28 | 52 | 80 |  |  |
|  | Null |  |  | 8 |  |  |
| Sex | Female | 19 | 22 | 41 | 0.9117 | 0.3397 |
|  | Male | 19 | 33 | 52 |  |  |
| TNM stage | I/II | 11 | 34 | 45 | 2.087 | 0.0369* |
|  | III/IV | 18 | 21 | 39 |  |  |
|  | Null |  |  | 9 |  |  |
| N stage | NO | 15 | 36 | 51 | 3.386 | 0.0658 |
|  | N1/N2 | 18 | 19 | 37 |  |  |
|  | Null |  |  | 5 |  |  |
| M | MO | 37 | 52 | 89 | 0.4351 | 0.5095# |
|  | M1 | 1 | 3 | 4 |  |  |
| Grade | I/II | 34 | 44 | 78 | 1.491 | 0.2221 |
|  | III(II-III) | 4 | 11 | 15 |  |  |
| ki67 | Negative(≤15%) | 16 | 22 | 38 | 0.02149 | 0.8834 |
|  | Positive(>15%) | 24 | 31 | 55 |  |  |
| p53 | Negative | 12 | 14 | 26 | 0.4185 | 0.5177 |
|  | Positive | 26 | 41 | 67 |  |  |
| MSH2 | Negative | 0 | 2 | 2 | 1.412 | 0.2347# |
|  | Positive | 38 | 53 | 91 |  |  |
| MSH6 | Negative | 3 | 2 | 5 | 0.8011 | 0.3708# |
|  | Positive | 35 | 53 | 88 |  |  |
| Surviving | Negative | 15 | 38 | 53 | 8.042 | 0.0046** |
|  | Positive | 23 | 17 | 40 |  |  |
|  |  |  |  |  |  |  |
|  | * The P value＜0.05 is regarded as statistically significant |  |  |  |  |  |
|  | #fisher-test |  |  |  |  |  |

**Table S3 Primers used for real-time RT-PCR.**

| **Gene** | **Sequences(5’ ￫ 3’)**  **(Forward)** | **Sequences(5’ ￫ 3’)**  **(Reverse)** | **Product length (bp)** | **GenBank accession number** |
| --- | --- | --- | --- | --- |
| METTL1 | GGCAACGTGCTCACTCCAA | CACAGCCTATGTCTGCAAACT | 192 | NM_023033 |
| CDK4 | ATGGCTACCTCTCGATATGAGC | CATTGGGGACTCTCACACTCT | 124 | NM_000075 |
| CDK6 | GCTGACCAGCAGTACGAATG | GCACACATCAAACAACCTGACC | 225 | NM_001145306 |
| C-MYC | GGCTCCTGGCAAAAGGTCA | CTGCGTAGTTGTGCTGATGT | 119 | NM_002467 |
| CDKN1A | TGTCCGTCAGAACCCATGC | AAAGTCGAAGTTCCATCGCTC | 139 | NM_078467 |
| CDC20 | GCACAGTTCGCGTTCGAGA | CTGGATTTGCCAGGAGTTCGG | 188 | NM_001255 |
| CHEK2-QPCR | TCTCGGGAGTCGGATGTTGAG | CCTGAGTGGACACTGTCTCTAA | 205 | NM_001005735 |
| FZR1 | CTGGAAGTACCCCTCCCTGA | AGACTCCTTTGTCGAACGGG | 166 | NM_001136197 |
| GAPDH | ACATCGCTCAGACACCATG | TGTAGTTGAGGTCAATGAAGGG | 143 | NM_001256799 |
| CHEK2-RIP | CTCTTGGAAGTGGTGCCTGT | ACATTGAGAGCTGGGTCTGC | 136 | NM_001005735 |
